# “So at least now I know how to deal with things myself, what I can do if it gets really bad again”—experiences with a long-term cross-sectoral advocacy care and case management for severe multiple sclerosis: a qualitative study

**DOI:** 10.1186/s12913-024-10851-1

**Published:** 2024-04-10

**Authors:** Anne Müller, Fabian Hebben, Kim Dillen, Veronika Dunkl, Yasemin Goereci, Raymond Voltz, Peter Löcherbach, Clemens Warnke, Heidrun Golla, on behalf of the COCOS-MS trial group represented by Martin Hellmich, Dirk Müller, Dorthe Hobus, Eckhard Bonmann, Franziska Schwartzkopff, Gereon Nelles, Gundula Palmbach, Herbert Temmes, Isabel Franke, Judith Haas, Julia Strupp, Kathrin Gerbershagen, Laura Becker-Peters, Lothar Burghaus, Martin Hellmich, Martin Paus, Solveig Ungeheuer, Sophia Kochs, Stephanie Stock, Thomas Joist, Volker Limmroth

**Affiliations:** 1 Department of Palliative Medicine, Faculty of Medicine and University Hospital, University of Cologne, Cologne, Germany; 2 Department of Neurology, Faculty of Medicine and University Hospital, University of Cologne, Cologne, Germany; 3 Center for Integrated Oncology Aachen Bonn Cologne Düsseldorf (CIO ABCD), University of Cologne, Cologne, Germany; 4 Center for Health Services Research, University of Cologne, Cologne, Germany; 5 German Society of Care and Case Management E.V. (DGCC), Münster, Germany

**Keywords:** Long-term, Cross-sectoral, Advocacy, Care and case management, Qualitative research, Patients, Caregivers, Health care specialist, Severe multiple sclerosis, Support

## Abstract

**Background:**

Persons with severe Multiple Sclerosis (PwsMS) face complex needs and daily limitations that make it challenging to receive optimal care. The implementation and coordination of health care, social services, and support in financial affairs can be particularly time consuming and burdensome for both PwsMS and caregivers. Care and case management (CCM) helps ensure optimal individual care as well as care at a higher-level. The goal of the current qualitative study was to determine the experiences of PwsMS, caregivers and health care specialists (HCSs) with the CCM.

**Methods:**

In the current qualitative sub study, as part of a larger trial, in-depth semi-structured interviews with PwsMS, caregivers and HCSs who had been in contact with the CCM were conducted between 02/2022 and 01/2023. Data was transcribed, pseudonymized, tested for saturation and analyzed using structuring content analysis according to Kuckartz. Sociodemographic and interview characteristics were analyzed descriptively.

**Results:**

Thirteen PwsMS, 12 caregivers and 10 HCSs completed interviews. Main categories of CCM functions were derived deductively: (1) gatekeeper function, (2) broker function, (3) advocacy function, (4) outlook on CCM in standard care. Subcategories were then derived inductively from the interview material. 852 segments were coded. Participants appreciated the CCM as a continuous and objective contact person, a person of trust (92 codes), a competent source of information and advice (on MS) (68 codes) and comprehensive cross-insurance support (128 codes), relieving and supporting PwsMS, their caregivers and HCSs (67 codes).

**Conclusions:**

Through the cross-sectoral continuous support in health-related, social, financial and everyday bureaucratic matters, the CCM provides comprehensive and overriding support and relief for PwsMS, caregivers and HCSs. This intervention bears the potential to be fine-tuned and applied to similar complex patient groups.

**Trial registration:**

The study was approved by the Ethics Committee of the University of Cologne (#20–1436), registered at the German Register for Clinical Studies (DRKS00022771) and in accordance with the Declaration of Helsinki.

**Supplementary Information:**

The online version contains supplementary material available at 10.1186/s12913-024-10851-1.

## Introduction

Multiple sclerosis (MS) is the most frequent and incurable chronic inflammatory and degenerative disease of the central nervous system (CNS). Illness awareness and the number of specialized MS clinics have increased since the 1990s, paralleled by the increased availability of disease-modifying therapies [1]. There are attempts in the literature for the definition of severe MS [2, 3]. These include a high EDSS (Expanded disability Status Scale [4]) of ≥ 6, which we took into account in our study. There are also other factors to consider, such as a highly active disease course with complex therapies that are associated with side effects. These persons are (still) less disabled, but may feel overwhelmed with regard to therapy, side effects and risk monitoring of therapies [5, 6].

Persons with severe MS (PwsMS) develop individual disease trajectories marked by a spectrum of heterogeneous symptoms, functional limitations, and uncertainties [7, 8] manifesting individually and unpredictably [9]. This variability can lead to irreversible physical and mental impairment culminating in complex needs and daily challenges, particularly for those with progressive and severe MS [5, 10, 11]. Such challenges span the spectrum from reorganizing biographical continuity and organizing care and everyday live, to monitoring disease-specific therapies and integrating palliative and hospice care [5, 10]. Moreover, severe MS exerts a profound of social and economic impact [9, 12–14]. PwsMS and their caregivers (defined in this manuscript as relatives or closely related individuals directly involved in patients’ care) often find themselves grappling with overwhelming challenges. The process of organizing and coordinating optimal care becomes demanding, as they contend with the perceived unmanageability of searching for, implementing and coordinating health care and social services [5, 15–17].

Case management (CM) proved to have a positive effect on patients with neurological disorders and/or patients with palliative care needs [17–24]. However, a focus on severe MS has been missed so far Case managers primarily function as: (1) *gatekeeper* involving the allocation of necessary and available resources to a case, ensuring the equitable distribution of resources; as (2) *broker* assisting clients in pursuing their interests, requiring negotiation to provide individualized assistance that aligns as closely as possible with individual needs and (3) *advocate* working to enhance clients’ individual autonomy, to advocate for essential care offers, and to identify gaps in care [25–29].

Difficulties in understanding, acting, and making decisions regarding health care-related aspects (health literacy) poses a significant challenge for 54% of the German population [30]. Additionally acting on a superordinate level as an overarching link, a care and case management (CCM) tries to reduce disintegration in the social and health care system [31, 32]. Our hypothesis is that a CCM allows PwsMS and their caregivers to regain time and resources outside of disease management and to facilitate the recovery and establishment of biographical continuity that might be disrupted due to severe MS [33, 34].

Health care specialists (HCSs) often perceive their work with numerous time and economic constraints, especially when treating complex and severely ill individuals like PwsMS and often have concerns about being blamed by patients when expectations could not be met [35, 36]. Our hypothesis is that the CCM will help to reduce time constraints and free up resources for specialized tasks.

To the best of our knowledge there is no long-term cross-sectoral and outreaching authority or service dedicated to assisting in the organization and coordination of the complex care concerns of PwsMS within the framework of standard care addressing needs in health, social, financial, every day and bureaucratic aspects. While some studies have attempted to design and test care programs for persons with MS (PwMS), severely affected individuals were often not included [37–39]. They often remain overlooked by existing health and social care structures [5, 9, 15].

The COCOS-MS trial developed and applied a long-term cross-sectoral CCM intervention consisting of weekly telephone contacts and monthly re-assessments with PwsMS and caregivers, aiming to provide optimal care. Their problems, resources and (unmet) needs were assessed holistically including physical health, mental health, self-sufficiency and social situation and participation. Based on assessed (unmet) needs, individual care plans with individual actions and goals were developed and constantly adapted during the CCM intervention. Contacts with HCSs were established to ensure optimal care. The CCM intervention was structured through and documented in a CCM manual designed for the trial [40, 41].

Our aim was to find out how PwsMS, caregivers and HCSs experienced the cross-sectoral long-term, outreaching patient advocacy CCM.

## Methods

This study is part of a larger phase II, randomized, controlled clinical trial “Communication, Coordination and Security for people with severe Multiple Sclerosis (COCOS-MS)” [41]. This explorative clinical trial, employing a mixed-method design, incorporates a qualitative study component with PwsMS, caregivers and HCSs to enrich the findings of the quantitative data. This manuscript focuses on the qualitative data collected between February 2022 and January 2023, following the Consolidated Criteria for Reporting Qualitative Research (COREQ) guidelines [42].

### Research team

Three trained authors AM, KD and FH (AM, female, research associate, M.A. degree in Rehabilitation Sciences; KD, female, researcher, Dr. rer. medic.; FH, male, research assistant, B.Sc. degree in Health Care Management), who had no prior relationship with patients, caregivers or HCSs conducted qualitative interviews. A research team, consisting of clinical experts and health services researchers, discussed the development of the interview guides and the finalized category system.

### Theoretical framework

Interview data was analyzed with the structuring content analysis according to Kuckartz. This method enables a deductive structuring of interview material, as well as the integration of new aspects found in the interview material through the inductive addition of categories in an iterative analysis process [43].

Sociodemographic and interview characteristics were analyzed descriptively (mean, median, range, SD). PwsMS, caregivers and HCSs were contacted by the authors AM, KD or FH via telephone or e-mail after providing full written informed consent. Participants had the option to choose between online interviews conducted via the GoToMeeting 10.19.0® Software or face-to-face. Peasgood et al. (2023) found no significant differences in understanding questions, engagement or concentration between face-to-face and online interviews [44, 45]. Digital assessments were familiar to participants due to pandemic-related adjustments within the trial.

**Fig. 1:**
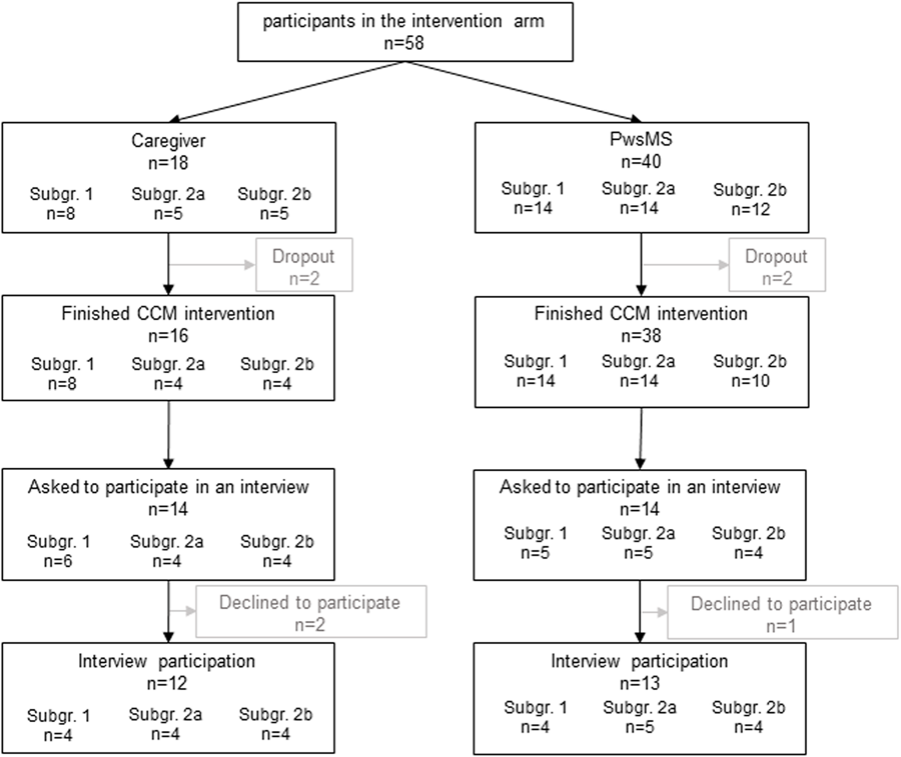
Flowchart of PwsMS and caregiver participation in the intervention group of the COCOS-MS trial. Patients could participate with and without a respective caregiver taking part in the trial. Therefore, number of caregivers does not correspond to patients. For detailed inclusion criteria see also Table 1 in Golla et al. [41]

**Fig. 2:**
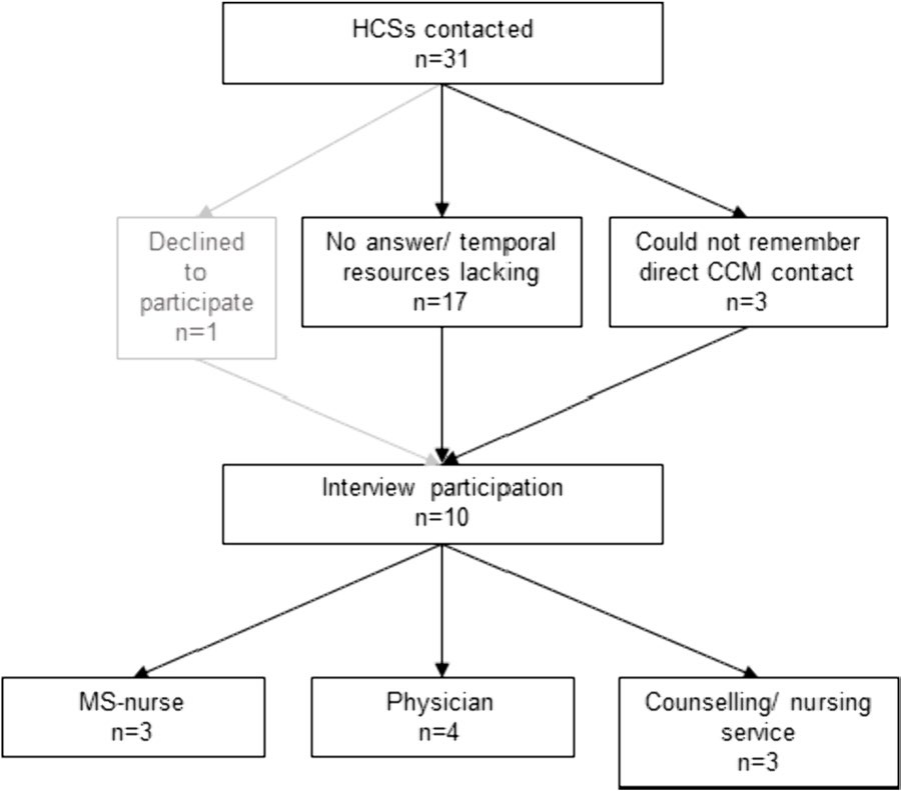
Flowchart of HCSs interview participation

Out of 14 PwsMS and 14 caregivers who were approached to participate in interviews, three declined to complete interviews, resulting in 13 PwsMS (5 male, 8 female) and 12 caregiver (7 male, 5 female) interviews, respectively (see Fig. 1). Thirty-one HCSs were contacted of whom ten (2 male, 8 female) agreed to be interviewed (see Fig. 2).

### Setting and data collection

Interviews were carried out where participants preferred, e.g. at home, workplace, online, and no third person being present. In total, we conducted 35 interviews whereof 7 interviews face-to-face (3 PwsMS, 3 caregivers, 1 HCS).

The research team developed a topic guide which was meticulously discussed with research and clinical staff to enhance credibility. It included relevant aspects for the evaluation of the CCM (see Tables 1 and 2, for detailed topic guides see Supplementary Material). Patient and caregiver characteristics (covering age, sex, marital status, living situation, EDSS (patients only), subgroup) were collected during the first assessment of the COCOS-MS trial and HCSs characteristics (age, sex, profession) as well as interview information (length and setting) were collected during the interviews. The interview guides developed for this study addressed consistent aspects both for PwsMS and caregivers (see Supplementary Material):

**Table 1:** Topic guide for PwsMS and their caregivers

• Opening question: What characterizes the work of a CCM for you?
• Block I: Functions and support of CCM
• Block II: Impact of the CCM intervention
• Block III: Communication and evaluation
• Closing questions: What was most important to you in your contact with the CCM? Do you have anything else to add we haven’t discussed yet?

**Table 2:** Topic guide for HCSs

• Opening question: What characterizes the work of a CCM for you?
• Block I: Functions and support of CCM
• Block II: Impact of the CCM intervention
• Block III: Outlook
• Closing questions: What was most important to you in your contact with the CCM? Do you have anything else to add we haven’t discussed yet?

For HCSs it contained the following guides:

Probing questions were asked to get more specific and in-depth information. Interviews were carried out once and recorded using a recording device or the recording function of the GoToMeeting 10.19.0® Software. Data were pseudonymized (including sensitive information, such as personal names, dates of birth, or addresses), audio files were safely stored in a data protection folder. The interview duration ranged from 11 to 56 min (mean: 23.9 min, SD: 11.1 min). Interviews were continued until we found that data saturation was reached. Audio recordings were transcribed verbatim by an external source and not returned to participants.

### Data analysis

Two coders (AM, FH) coded the interviews. Initially, the first author (AM) thoroughly reviewed the transcripts to gain a sense of the interview material. Using the topic guide and literature, she deductively developed a category system based on the primary functions of CM [25–29]. Three interviews were coded repeatedly for piloting, and inductive subcategories were added when new themes emerged in the interview material. This category system proved suitable for the interview material. The second coder (FH) familiarized himself with the interview material and category system. Both coders (AM, FH) independently coded all interviews, engaging in discussions and adjusting codes iteratively. The finalized category system was discussed and consolidated in a research workshop and within the COCOS-MS trial group and finally we reached an intercoder agreement of 90% between the two coders AM and FH, computed by the MAXQDA Standard 2022® software.

We analyzed sociodemographic and interview characteristics using IBM SPSS Statistics 27® and Excel 2016®. Transcripts were managed and analyzed using MAXQDA Standard 2022®.

Participants were provided with oral and written information about the trial and gave written informed consent. Ethical approvals were obtained from the Ethics Committee of the University of Cologne (#20–1436). The trial is registered in the German Register for Clinical Studies (DRKS) (DRKS00022771) and is conducted under the Declaration of Helsinki.

## Results

### Characteristics of participants and interviews

PwsMS participating in an interview were mainly German (84.6%), had a mean EDSS of 6.8 (range: 6–8) and MS for 13.5 years (median: 14; SD: 8.1). For detailed characteristics see Table 3.

**Table 3:** Interview and sociodemographic characteristics

	Total n (%)	Caregiver n (%)	HCS n (%)	PwsMS n (%)
**Sex**				
*Female*	21 (60)	5 (41.6)	8 (80.0)	8 (61.5)
*Male*	14 (40)	7 (58.3)	2 (20.0)	5 (38.5)
**Age**				
Mean	54.2	57.3	50.8	53.9
*SD*	9.5	8.4	6.7	11.7
**Interview duration [min]**				
Mean	23.9	27.4	24.7	20.1
*SD*	11.1	12.5	11.7	8.7
**Interview location**				
*Online*	28 (80.0)	9 (75.0)	9 (90.0)	10 (76.9)
*Face-to-face*	7 (20.0)	3 (25.0)	1 (10.0)	3 (23.1)
**Relationship between participants**				
*Partner*		9 (75.0)		7 (53.8)
*Parent*		3 (25.0)		
*No caregiver included*				6 (46.2)
**Living situation**^a^				
*Alone*				5
*Partner*		12		7
*Child*		5		2
*Patient*		1		
**Children**				
*Yes*		10 (83.3)		7 (53.8)
*No*		2 (16.7)		6 (46.2)
**Country of origin**				
*Germany*				11 (84.6)
*Poland*				1 (7.7)
*Hungary*				1 (7.7)
**Subgroup**^b^				
*Subgroup 1*				4 (30.8)
*Subgroup 2a*				5 (38.5)
*Subgroup 2b*				4 (30.8)
**EDSS at the time of inclusion into the COCOS-MS study**				
*Mean*				6.8
*Median*				7.0
*SD*				0.7
*Min*				6.0
*Max*				8.0
**Disease duration in years**				
*Mean*				13.5
*Median*				14
*SD*				8.1
*Min*				3
*Max*				26
**Highest school qualification (german system)**				
*Abitur (higher education entrance qualification (A-levels))*		3 (25)		4 (30.8)
*Fachabitur (subject-related entrance qualification)*		1 (8.3)		5 (38.5)
*Mittlere Reife (intermediate secondary school-leaving certificate (O-levels))*		3 (25)		2 (15.4)
*Hauptschulabschluss (main school qualification)*		5 (41.7)		2 (15.4)
**Marital status**				
*Single*		1 (8.3)		3 (23.1)
*Married/ partnership*		10 (83.3)		7 (53.8)
*Divorced*		1 (8.3)		2 (15.4)
*Widowed*				1 (7.7)

^a^Multiple answers possible

^b^For detailed inclusion criteria see Table 1 in Golla et al. [41]

Most of the interviewed caregivers (9 caregivers) were the partners of the PwsMS with whom they lived in the same household. For further details see Table 3.

HCSs involved in the study comprised various professions, including MS-nurse (3), neurologist (2), general physician with further training in palliative care (1), physician with further training in palliative care and pain therapist (1), housing counselling service (1), outpatient nursing service manager (1), participation counselling service (1).

### Structuring qualitative content analysis

**Fig. 3:**
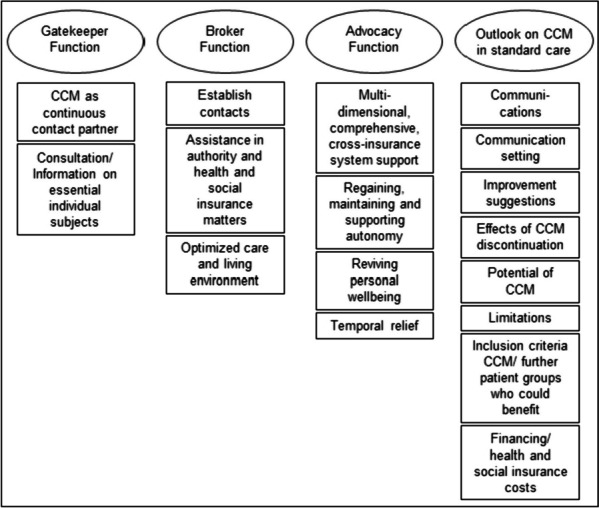
Category system including main and subcategories of the qualitative thematic content analysis

The experiences of PwsMS, caregivers and HCSs were a priori deductively assigned to four main categories: (1) gatekeeper function, (2) broker function, (3) advocacy function [25–29] and (4) Outlook on CCM in standard care, whereas the subcategories were developed inductively (see Fig. 3).

The most extensive category, housing the highest number of codes and subcodes, was the “*Outlook on CCM in standard care*” (281 codes). Following this, the category “*Advocacy Function*” contained 261 codes. The “*Broker Function*” (150 codes) and the “*Gatekeeper Function*” (160 codes) constituted two smaller categories. The majority of codes was identified in the caregivers’ interviews, followed by those of PwsMS (see Table 4). Illustrative quotes for each category and subcategory can be found in Table 5.

**Table 4:** Frequency of codes and subcodes in the three groups

*Code*	*PwsMS*	*Caregiver*	*HCS*	*Total*
**Gatekeeper Function**	**59**	**75**	**26**	**160**
CCM as continuous contact partner	41	33	18	92
Consultation/Information on essential individual subjects	18	42	8	68
**Broker Function**	**44**	**63**	**43**	**150**
Establish contacts	22	24	18	64
Assistance in authority and health and social insurance matters	10	22	7	39
Optimized care and living environment	12	17	18	47
**Advocacy Function**	**103**	**115**	**43**	**261**
Multidimensional, comprehensive, cross-insurance system support	43	70	15	128
Regaining, maintaining and supporting autonomy	25	11	3	39
Reviving personal wellbeing	17	8	2	27
Temporal relief	18	26	23	67
**Outlook on CCM in standard care**	**84**	**81**	**116**	**281**
Communications	9	7	2	18
Communication setting	29	10	5	44
Improvement suggestions	10	13	21	44
Effects of CCM discontinuation	10	9	3	22
Potential of CCM	11	27	20	58
Limitations	7	8	13	28
Inclusion criteria CCM/ further patient groups who could benefit	6	7	38	51
Financing/health and social insurance costs	2	0	14	16
*Total codes*	*290*	*334*	*228*	*852*
*Total interviews*	*13*	*12*	*10*	*35*

**Table 5:** Illustrative quotes for categories and subcategories of the qualitative content analysis

Code	Subcode	Illustrative quote
Gatekeeper Function	CCM as continuous contact partner	“Yes, I could—I felt like I could share my worries and my questions and my concerns, but also my wishes with somebody neutral, who would judge as little as possible.” (Patient 39, Pos. 91)
	Consultation/ Information on essential individual subjects	“[…] she would advise with, with—with what is possible regarding purchases or even like bureaucratically. So yeah, with anything.” (Patient 14, Pos. 5)
Broker Function	Establish contacts	“So for instance, < NAME CM > instantly got me a fully accessible gynecologist. And I immediately found a therapist, which is great. That would have not been possible without the help, I think, right. Because it’s not that easy, right.” (Patient 25, Pos. 9)
	Assistance in authority and health and social insurance maters matters	“She once helped us with the pension application, when we couldn’t figure it out.” (Caregiver 45, Pos. 7)
	Optimized care and living environment	“[…] this caused the patient to achieve the goal pretty quickly, like—so in our case, the diagnosis and that I think, for one, made it quicker and speed up the choice of therapy based on that diagnosis. So it was somewhat accelerated and maybe the wait was shortened and stress was prevented for the patient, who would have had to request several places and possibly would have not reached the goal at all and depending on the patient, would have given up.” (HCS 08, Pos. 51)
Advocacy Function	Multidimensional, comprehensive, cross-insurance system support	“So in principle, for me or for us, so for me and my partner, it was a continual kind of support that gave some sort of orientation.” (Caregiver 15, Pos.11)
	Regaining, maintaining and supporting autonomy	“So at least now I know how to deal with things myself. What I can do if it gets really bad again and that was not true before and my doctor had kind of no more ideas except giving me cortisone over and over and, I don’t know, every six weeks five grams of cortisone, not sure if that’s great on the long run (laughs). So therefore it’s really good that I now have an alternative.” (Pat. 14, Pos.19)
	Reviving personal wellbeing	“[…] the relief thanks to < NAME CM > , that improved quality of life. I mean, which makes perfect sense.” (Caregiver 45, Pos. 72)
	Temporal relief through CCM intervention	“But you feel like you’re in good hands. That relieves you, that relieved my wife and also myself, yes.” (Caregiver 45, Pos. 184)
Outlook on CCM in standard care	Communications	“So the communication was always, yeah, friendly, helpful, so I did feel it to be nice communication.” (Patient 34, Pos.91)
	Communication setting	“So the phone calls eventually—I found them sufficient, because the better you know a person, the more you really know how they operate or—and vice versa. But I always liked it, I think it’s important to see each other once a month. Especially in the beginning, but I think—I think the three-week-thing with phone calls, once a week home visits is right.” (Patient 39, Pos.121)
	Improvement suggestions	“Yeah and also, maybe especially with patients who are severely affected, and yeah. Needing good and more multifaceted support, yeah, then some sort of feedback would—would indeed be a good thing.” (HCS 10, Pos.28)
	Effects of CCM discontinuation	“[…] when other things need to be dealt with, but like I said, there is a lack of somebody with whom you’re like: ‘You know what, I’ll just call them, maybe they have an idea or something.’” (Patient 22, Pos.60)
	Potential of CCM	“Yeah, < NAME CM > really did a great job and it was truly pleasant on a human level and also professionally and it really helped, like it was a really great thing, yes. We would have needed that two years earlier (laughs).” (Patient 22, Pos.30)
	Limitations	“[…] a thing that didn’t work, which wasn’t her fault, was occupational therapy. Both ladies made such an effort but unfortunately it didn’t work because there is a lack of staff.” (Caregiver 77, Pos. 25)
	Inclusion criteria CCM/ further patient groups who could benefit	“You probably have to select carefully who the prototypical patient would be that needs it. Right, because there are close relatives or even patients themselves who make an active effort to get informed and support themselves really well.” (HCS 08, Pos. 47)
	Financing/health and social insurance costs	“I think that, from—like, for a specific clientele of patients I can imagine that partly the costs could be better distributed or rather, how can I say this, be better allocated.” (HCS 08, Pos. 47)

### Persons with severe multiple sclerosis

In the *gatekeeper function* (59 codes), PwsMS particularly valued the *CCM as a continuous contact person*. They appreciated the CCM as a person of trust who was reliably accessible throughout the intervention period. This aspect, with 41 codes, held significant importance for PwsMS.

Within the *broker function* (44 codes), *establishing contact* was most important for PwsMS (22 codes). This involved the CCM as successfully connecting PwsMS and caregivers with physicians and therapists, as well as coordinating and arranging medical appointments, which were highly valued. *Assistance in authority and health and social insurance matters* (10 codes) was another subcategory, where the CCM encompassed support in communication with health insurance companies, such as improving the level of care, assisting with retirement pension applications, and facilitating rehabilitation program applications. *Optimized care (12 codes)* resulted in improved living conditions and the provision of assistive devices through the CCM intervention.

The *advocacy function* (103 codes) emerged as the most critical aspect for PwsMS, representing the core of the category system. PwsMS experienced *multidimensional, comprehensive, cross-insurance system support* from the CCM. This category, with 43 statements, was the largest within all subcategories. PwsMS described the CCM as addressing their concerns, providing help, and assisting with the challenges posed by the illness in everyday life. The second-largest subcategory, *regaining, maintaining and supporting autonomy* (25 codes), highlighted the CCM’s role in supporting self-sufficiency and independence. *Reviving personal wellbeing* (17 codes) involved PwsMSs’ needs of regaining positive feelings, improved quality of life, and a sense of support and acceptance, which could be improved by the CCM. *Temporal relief* (18 codes) was reported, with the CCM intervention taking over or reducing tasks.

Within the *outlook on CCM in standard care* (84 codes), eight subcategories were identified. *Communications* was described as friendly and open (9 codes), with the *setting of communication* (29 codes) including the frequency of contacts deemed appropriate by the interviewed PwsMS, who preferred face-to-face contact over virtual or telephone interactions. *Improvement* suggestions *for CCM* (10 codes) predominantly revolved around the desire for the continuation of the CCM beyond the trial, expressing intense satisfaction with the CCM contact person and program. PwsMS rarely wished for better cooperation with the CCM. With respect to *limitations* (7 codes), PwsMS distinguished between individual limitations (e.g. when not feeling ready for using a wheelchair) and overriding structural limitations (e.g. unsuccessful search for an accessible apartment despite CCM support). Some PwsMS mentioned needing the CCM earlier in the course of the disease and believed it would beneficial for anyone with a chronic illness (6 codes).

### Caregivers

In the *gatekeeper function* (75 codes), caregivers highly valued the CCM as a *continuous contact partner* (33 codes). More frequently than among the PwsMS interviewed, caregivers valued the *CCM as a source of consultation/ information on essential individual subjects* (42 codes). The need for basic information about the illness, its potential course, treatment and therapy options, possible supportive equipment, and basic medical advice/ information could be met by the CCM.

Within the *broker function* (63 codes), caregivers primarily experienced the subcategory *establish contacts* (24 codes). They found the CCM as helpful in establishing and managing contact with physicians, therapists and especially with health insurance companies. In the subcategory *assistance in authority and health and social insurance matters* (22 codes), caregivers highlighted similar aspects as the PwsMS interviewed. However, there was a particular emphasis on assistance with patients' retirement matters. Caregivers also valued the optimization of *patients’ care and living environment* (17 codes) in various life areas during the CCM intervention, including improved access to assistive devices, home modification, and involvement of a household support and/ or nursing services.

The *advocacy function,* with 115 codes, was by far the broadest category*.* The subcategory *multidimensional, comprehensive, cross-insurance system support* represented the largest subcategory of caregivers, with 70 statements. In summary, caregivers felt supported by the CCM in all domains of life. *Regaining, maintaining and supporting autonomy* (11 codes) and *reviving personal wellbeing* (8 codes) in the form of an improved quality of life played a role not only for patients but also for caregivers, albeit to a lower extend. Caregivers experienced *temporal relief* (26 codes) as the CCM undertook a wide range of organizational tasks, freeing up more needed resources for their own interests.

For the *Outlook on CCM in standard care*, caregivers provided various suggestions (81 codes). Similar to PwsMS, caregivers felt that setting (home based face-to-face, telephone, virtual) and frequency of contact were appropriate (10 codes, *communication setting*) *and communications* (7 codes) were recognized as open and friendly. However, to avoid conflicts between caregiver and PwsMS, caregivers preferred meeting the CCM separately from the PwsMS in the future. Some caregivers wished the CCM to specify all services it might offer at the beginning, while others emphasized not wanting this. Like PwsMS, caregivers criticized the CCM intervention being (trial-related) limited to one year, regardless of whether further support was needed or processes being incomplete (13 codes, *improvement suggestions*). After the CCM intervention time had expired, the continuous contact person and assistance were missed and new problems had arisen and had to be managed with their own resources again (9 codes, *effects of CCM discontinuation*), which was perceived as an exhausting or unsolvable endeavor. Caregivers identified analogous *limitations* (8 codes), both individual and structural. However, the largest subcategory, was the experienced *potential of CCM* (27 codes), reflected in extremely high satisfaction with the CCM intervention. Like PwsMS, caregivers regarded severe chronically ill persons in general as *target groups for a CCM* (7 codes) and would implement it even earlier, starting from the time of diagnosis. They considered a CCM to be particularly helpful for patients without caregivers or for caregivers with limited (time) resources, as it was true for most caregivers.

### Health care specialists

In the *gatekeeper function* (26 codes) HCSs particularly valued the *CCM as a continuous contact partner* (18 codes). They primarily described their valuable collaboration with the CCM, emphasizing professional exchange between the CCM and HCSs.

Within the *broker function* (43 codes), the CCM was seen as a connecting link between patients and HCSs, frequently establishing *contacts* (18 codes). This not only improved optimal care on an individual patient level (case management) but also at a higher, superordinate care level (care management). HCSs appreciated the *optimized care and living environment* (18 codes) for PwsMS, including improved medical and therapeutic access and the introduction of new assistive devices. The CCM was also recognized as providing *assistance in authority and health and social matters* (7 codes) for PwsMS and their caregivers.

In the *advocacy function* (43 codes), HCSs primarily reported *temporal relief* through CCM intervention (23 codes). They experienced this relief, especially as the CCM provided *multidimensional, comprehensive, and cross-insurance system support* (15 codes) for PwsMS and their caregivers. Through this support, HCSs felt relieved from time intensive responsibilities that may not fall within their area of expertise, freeing up more time resources for their actual professional tasks.

The largest category within the HCSs interviews was the *outlook on CCM in standard care* (116 codes). In the largest subcategory, HCSs made suggestions for *further patient groups who could benefit* (38 codes) from a CCM. Chronic neurological diseases like neurodegenerative diseases (e.g. amyotrophic lateral sclerosis), typical and atypical Parkinson syndromes were mentioned. HCSs considered the enrollment of the CCM directly after the diagnosis of these complex chronic diseases. Additionally, chronic progressive diseases in general or oncological diseases, which may also run chronically, were regarded worthwhile for this approach. HCSs also provided suggestions regarding *improvement* (21 codes). They wished e.g. for information or contact when patients were enrolled to the CCM, regular updates, exchange and collaborative effort. On the other hand, HCSs reported, that their suggestions for improvement would hardly be feasible due to their limited time resources. Similar to patients and caregivers, HCSs experienced structural limits (13 codes), which a CCM could not exceed due to overriding structural limitations (e.g. insufficient supply of (household) aids, lack of outreach services like psychotherapists, and long processing times on health and pension insurers' side). HCSs were also asked about their opinions on *financial resources* (14 codes) of a CCM in standard care. All interviewed HCSs agreed that CCM would initially cause more costs for health and social insurers, but they were convinced of cost savings in the long run. HCSs particularly perceived the *potential of the CCM* (20 codes) through the feedback of PwsMS, highlighting the trustful relationship enabling individualized help for PwsMS and their caregivers.

## Discussion

### Persons with severe multiple sclerosis and their caregivers

The long-term cross-sectoral CCM intervention implemented in the COCOS-MS trial addressed significant unmet needs of PwsMS and their caregivers which previous research revealed as burdensome and hardly or even not possible to improve without assistance [5, 6, 9, 10, 33, 35, 46]. Notably, the CCM service met the need for a reliable, continuous contact partner, guiding patients through the complexities of regulations, authorities and the insurance system. Both, PwsMS and their caregivers highly valued the professional, objective perspective provided by the CCM, recognizing it as a source of relief, support and improved care in line with previous studies [37, 47]. Caregivers emphasized the CCM’s competence in offering concrete assistance and information on caregiving and the fundamentals of MS, including bureaucratic, authority and insurances matters. On the other hand, PwsMS particularly appreciated the CCMs external reflective and advisory function, along with empathic social support tailored to their individual concerns. Above all, the continuous partnership of trust, available irrespective of the care sector, was a key aspect that both PwsMS and their caregivers highlighted. This consistent support was identified as one of the main components in the care of PwsMS in previous studies [5, 33, 35].

As the health literacy is inadequate or problematic for 54% of the German population and disintegration in the health and social care system is high [30–32], the CCM approach serves to enhance health literacy and reduce disintegration of PwsMS and their caregivers by providing cross-insurance navigational guidance in the German health and social insurance sector on a superordinate level. Simultaneously PwsMS and caregivers experienced relief and gained more (time) resources for all areas of life outside of the disease and its management, including own interests and establishing biographical continuity. This empowerment enables patients to find a sense of purpose beyond their illness, regain autonomy, and enhance social participation, reducing the feeling of being a burden to those closest to them. Such feelings are often experienced as burdensome and shameful by PwsMS [6, 48–50]. Finding a sense of purpose beyond the illness also contributes to caregivers perceiving their loved ones not primarily as patient but as individuals outside of the disease, reinforcing valuable relationships such as partners, siblings, or children, strengthening emotional bonds. These factors are also highly relevant and well-documented in a suicide-preventive context, as the suicide rate is higher in persons diagnosed with neurological disorders [19, 51–60] and the feeling of being a burden to others, loss of autonomy, and perceived loss of dignity are significant factors in patients with severe chronic neurological diseases for suicide [50, 57].

### Health care specialists

The temporal relief experienced by the CCM was particularly significant for HCSs and did not only improve the satisfaction of HCSs but also removed unfulfilled expectations and concerns about being blamed by patients when expectations could not be met, which previous studied elaborated [35, 36]. Moreover, the CCM alleviated the burden on HCSs by addressing patients’ concerns, allowing them to focus on their own medical responsibilities. This aspect probably reduced the dissatisfaction that arises when HCSs are expected to address issues beyond their medical expertise, such as assistive devices, health and social insurance, and the organization and coordination of supplementary therapies, appointments, and contacts [35, 36, 61]. Consequently, the CCM reduced difficulties of HCSs treating persons with neurological or chronical illnesses, which previous research identified as problematic.

HCSs perceive their work as increasingly condensed with numerous time and economic constraints, especially when treating complex and severely ill individuals like PwsMS [36]. This constraint was mentioned by HCSs in the interviews and was one of the main reasons why they were hesitant to participate in interviews and may also be an explanation for a shorter interview duration than initially planned in the interview guides. The CCM’s overarching navigational competence in the health and social insurance system was particularly valued by HCSs. The complex and often small-scale specialties in the health and social care system are not easily manageable or well-known even for HCSs, and dealing with them can exceed their skills and time capacities [61]. The CCM played a crucial role in keeping (temporal) resources available for what HCSs are professionally trained and qualified to work on. However, there remains a challenge in finding solutions to the dilemma faced by HCSs regarding their wish to be informed about CCM procedures and linked with each other, while also managing the strain of additional requests and contact with the CCM due to limited (time) resources [62]. Hudon et al. (2023) suggest that optimizing time resources and improving exchange could involve meetings, information sharing via fax, e-mail, secure online platforms, or, prospectively, within the electronic patient record (EPR). The implementation of an EPR has shown promise in improving the quality of health care and time resources, when properly implemented [63, 64]. The challenge lies ineffective information exchange between HCSs and CCM for optimal patient care. The prospect of time saving in the long run and at best for a financial incentive, e.g., when anchoring in the Social Security Code, will help best to win over the HCSs.If this crucial factor can be resolved, there is a chance that HCSs will thoroughly accept the CCM as an important pillar, benefiting not only PwsMS but also other complex patient groups, especially those with long-term neurological or complex oncological conditions that might run chronically.

### Care and case management and implications for the health care system

The results of our study suggest that the cross-sectoral long-term advocacy CCM in the COCOS-MS trial, with continuous personal contacts at short intervals and constant reevaluation of needs, problems, resources and goals, is highly valued by PwsMS, caregivers, and HCSs. The trial addresses several key aspects that may have been overlooked in previous studies which have shown great potential for the integration of case management [17, 47, 62, 65, 66]. However, they often excluded the overriding care management, missed those patient groups with special severity and complexity who might struggle to reach social and health care structures independently or the interventions were not intended for long-term [22, 37]. Our results indicate that the CCM intervention had a positive impact on PwsMS and caregivers as HCSs experienced them with benefits such as increased invigoration, reduced demands, and enhanced self-confidence. However, there was a notable loss experienced by PwsMS and caregivers after the completion of the CCM intervention, even if they had stabilized during the intervention period. The experiences of optimized social and health care for the addressed population, both at an individual and superordinate care level, support the integration of this service into standard care. Beyond the quantitatively measurable outcomes and economic considerations reported elsewhere [16, 20, 21], our results emphasize the importance of regaining control, self-efficacy, self-worth, dignity, autonomy, and social participation. These aspects are highlighted as preventive measures in suicidal contexts, which is particularly relevant for individuals with severe and complex illnesses [19, 50, 53–60]. Our findings further emphasize the societal responsibilities to offer individuals with severe and complex illnesses the opportunity to regain control and meaningful aspects of life, irrespective of purely economic considerations. This underscores the need for a comprehensive evaluation that not only takes into account quantitative measures but also the qualitative aspects of well-being and quality of life when making recommendations of a CCM in standard care.

The study by J. Y. Joo and Huber (2019) highlighted that CM interventions aligned with the standards of the Case Management Society of America varied in duration, ranging from 1 month to 15.9 years, and implemented in community- or hospital-based settings. However, they noted a limitation in understanding how CM processes unfold [67]. In contrast, our trial addressed this criticism by providing transparent explanations of the CCM process, which also extends to a superordinate care management [40, 41]. Our CCM manual [40] outlines a standardized and structured procedure for measuring and reevaluating individual resources, problems, and unmet needs on predefined dimensions. It also identifies goals and actions at reducing unmet needs and improving the individual resources of PwsMS and caregivers. Importantly, the CCM manual demonstrates that the CCM process can be structured and standardized, while accounting for the unique aspects of each individual’s serious illness, disease courses, complex needs, available resources, and environmental conditions. Furthermore, the adaptability of the CCM manual to other complex chronically ill patient groups suggests the potential for a standardized approach in various health care settings. This standardized procedure allows for consistency in assessing and addressing the individual needs of patients, ensuring that the CCM process remains flexible while maintaining a structured and goal-oriented framework.

The discussion about the disintegration in the social and health care system and the increasing specialization dates back to 2009 [31, 32]. Three strategies were identified to address this issue: (a) “driver-minimizing” [Treiberminimierende], (b) “effect-modifying” [Effektmodifizierende] and (c) “disintegration-impact-minimizing” [Desintegrationsfolgenminimierende] strategies. “Driver-minimizing strategies” involve comprehensive and radical changes within the existing health and social care system, requiring political and social pursuit. “Disintegration-impact-minimizing strategies” are strategies like quality management or tele-monitoring, which are limited in scope and effectiveness. “Effect-modifying strategies”, to which CCM belongs, acknowledges the segmentation within the system but aims to overcome it through cooperative, communicative, and integrative measures. CCM, being an “effect-modifying strategy”, operates the “integrated segmentation model” [Integrierte Segmentierung] rather than the “general contractor model” [Generalunternehmer-Modell] or “total service provider model” [Gesamtdienstleister-Modell] [31, 32]. In this model, the advantage lies in providing an overarching and coordinating service to link different HCSs and services cross-sectorally. The superordinate care management aspect of the CCM plays a crucial role in identifying gaps in care, which is essential for future development strategies within the health and social care system. It aims to find or develop (regional) alternatives to ensure optimal care [17, 23, 24, 68, 69], using regional services of existing health and social care structures. Therefore, superordinate care management within the CCM process is decisive for reducing disintegration in the system.

### Strengths and limitations

The qualitative study results of the explorative COCOS-MS clinical trial, which employed an integrated mixed-method design, provide valuable insights into the individual experiences of three leading stakeholders: PwsMS, caregivers and HCSs with a long-term cross-sectoral CCM. In addition to in-depth interviews, patient and caregiver reported outcome measurements were utilized and will be reported elsewhere. The qualitative study’s strengths include the inclusion of patients who, due to the severity of their condition (e.g. EDSS mean: 6.8, range: 6–8, highly active MS), age (mean: 53.9 years, range: 36–73 years) family constellations, are often underrepresented in research studies and often get lost in existing social and health care structures. The study population is specific to the wider district region of Cologne, but the broad inclusion criteria make it representative of severe MS in Germany. The methodological approach of a deductive and inductive structuring content analysis made it possible to include new findings into an existing theoretical framework.

However, the study acknowledges some limitations. While efforts were made to include more HCSs, time constraints on their side limited the number of interviews conducted and might have biased the results. Some professions are underrepresented in the interviews. Complex symptoms (e.g. fatigue, ability to concentrate), medical or therapeutic appointments and organization of the everyday live may have been reasons for the patients’ and caregivers’ interviews lasting shorter than initially planned.

The provision of functions of a CCM, might have pre-structured the answers of the participants.

## Conclusion

At current, there is no support system for PwsMS, their caregivers and HCSs that addresses their complex and unmet needs comprehensively and continuously. There are rare qualitative insights of the three important stakeholders: PwsMS, caregivers and HCSs in one analysis about a supporting service like a CCM. In response to this gap, we developed and implemented a long-term cross-sectoral advocacy CCM and analyzed it qualitatively. PwsMS, their caregivers and HCSs expressed positive experiences, perceiving the CCM as a source of relief and support that improved care across various aspects of life. For patients, the CCM intervention resulted in enhanced autonomy, reviving of personal wellbeing and new established contacts with HCSs. Caregivers reported a reduced organizational burden and felt better informed, and HCSs experienced primarily temporal relief, allowing them to concentrate on their core professional responsibilities. At a higher level of care, the study suggests that the CCM contributed to a reduction in disintegration within the social and health care system.

The feedback from participants is seen as valuable for adapting the CCM intervention and the CCM manual for follow-up studies, involving further complex patient groups such as neurological long-term diseases apart from MS and tailoring the duration of the intervention depending on the complexity of evolving demands.

### Supplementary Information

**Supplementary Material 1. **

## Data Availability

Generated and/or analyzed datasets of participants are available from the corresponding author on reasonable request to protect participants. Preliminary partial results have been presented as a poster during the EAPC World Congress in June 2023 and the abstract has been published in the corresponding abstract booklet [70].

## Abbreviations

ALS: Amyotrophic lateral sclerosis
CCM: Care and case management
CM: Case management
CNS: Central nervous system
COCOS-MS: Communication, Coordination and security for people with multiple sclerosis
COREQ: Consolidated criteria for reporting qualitative research
DRKS: German register for clinical studies
EDSS: Extended disability status scale
EPR: Electronic patient record
HCS: Health care specialists
QOL: Quality of life
MS: Multiple sclerosis
PwsMS: Persons with severe multiple sclerosis
