# Supplementary material for: “So at least now I know how to deal with things myself, what I can do if it gets really bad again”—experiences with a long-term cross-sectoral advocacy care and case management for severe multiple sclerosis: a qualitative study
Source: BMC Health Serv Res. 2024 Apr 10;24:453. doi: 10.1186/s12913-024-10851-1 (PMC11007872; doi:10.1186/s12913-024-10851-1)
Supplement: Supplementary file 1 — Supplementary Material 1. [file 12913_2024_10851_MOESM1_ESM.zip › Interview guide_HCS.pdf]

Interview guide for focus group interviews (or individual interviews) with health care specialists within the trial "**Co**mmunication, **Co**ordination and **S**ecurity for Persons with **M**ultiple **S**clerosis" (COCOS-MS)

**Introduction**
Within the COCOS-MS study, you were in contact with the study case and care manager. Today I would like to speak with you about your personal experiences with and assessments of the care and case manager and find out to what extent you think the care of MS patients with complex needs and their caregivers can be improved by consulting a CCM. This is very important for the comprehensive evaluation and further development of such a service.

**Transition question** (Directing to the topic) (5minutes)
Based on your own **personal experience** with the care and case manager, what would you say characterizes the work of a CCM to you?

**Key questions** (15minutes each)		
**Opening question / narrative prompt**	**Memo for possible follow-up questions – only to be asked in case they are not addressed prior**	**Specific questions - ask in this wording**
**Block I: Functions and support of CCM**	- Initiation and coordination of necessary services	- Please describe to me what influence, if any, the CCM had on establishing contact

By definition, the work of a CCM can be divided into three key functions. The first is the social advocacy function that aims to support people with safeguarding their own interests and managing their own affairs independently. In the so-called broker function, the care and case manager sees themselves as an intermediary between patients and the providers of care services, i.e. access to the required services is created. The gatekeeper function is the third characteristic of care and case management. In this role, the care and case manager is responsible for assessing a patient's suitability and eligibility for access to care services. Based on this summary and your own previous experience with CCM studies, what would you add to or adjust about the work and functions of a CCM?	- Cross-sectoral approach to dealing with the handling of needs<br>- Cooperation at specialist level?<br>- Adoption of process control<br>- Special role / innovative aspect of CCM in the context of action<br>- Relief? Also for patients and their caregivers	between you and patients or between you and other service providers.<br>- Where did the care and case manager fail or what would need to change for the initiation and coordination of your services to improve? Can you give specific examples?<br>- In what instances did you have the impression that you needed more support from the CCM to provide comprehensive patient-oriented care?<br>- Please describe to me which tasks / activities the CCM has taken on that no one else in the healthcare sector has done so far. Can you give me examples?<br>- To what extent and in what way do the activities of the CCM relieve you in your day-to-day work? In your opinion, what aspects remained open / unfulfilled?<br>If the participant mentions relief for patients and their caregivers:<br>- You just reported that, can you say a little more about this? To what extent and in what respect do the patients and their caregivers experience relief from the CCM as a result of this or any other activities (if applicable, give examples such as regulating social law matters, finding suitable professional helpers, arranging appointments, higher-level exchange of information)?
**Block II: Impact of the CCM intervention**<br><br>If you imagine patient care with and without CCM, what is the most significant difference to you, if there is one?	- Benefit?<br>- Improvement?<br>- No change?<br>- Effects on patients?<br>- Burden due to additional actor?	- To what extent has your day-to-day work in patient care changed (positively or negatively) as a result of the CCM?<br>- What effects (positive or negative) have you been able to perceive as a result of the CCM as an addition to your everyday

		work life and your environment, e.g. on your patients / colleagues? Please describe them.<br>- To what extent does the CCM contribute to maintaining and developing the self-empowerment and self-efficacy of your patients?<br><br>If the participants cannot think of anything, ask specifically about the aspects in the memo
**Block III: Outlook**<br><br>The project management consciously decided to investigate the effects of a CCM study using the exemplary population of patients with severe MS. For which other patient groups can a CCM be helpful?	- Outstanding role of cross-sectoral CCM for patients with complex chronic neurological conditions in general<br>- CCM as a health insurance benefit?	- Can you elaborate a little more on this? How could a CCM be helpful for the patient group you mentioned?<br>- To what extent do you think it makes sense for a cross-sectoral CCM to become a health insurance benefit in the future (for the patients you mentioned)

**Closing Question** (10min)
What was most important to you about your contact with the care and case manager?<br>Is there anything else about care and case management that has not been mentioned but that you would still like to comment on?
