# Supplementary material for: “So at least now I know how to deal with things myself, what I can do if it gets really bad again”—experiences with a long-term cross-sectoral advocacy care and case management for severe multiple sclerosis: a qualitative study
Source: BMC Health Serv Res. 2024 Apr 10;24:453. doi: 10.1186/s12913-024-10851-1 (PMC11007872; doi:10.1186/s12913-024-10851-1)
Supplement: Supplementary file 1 — Supplementary Material 1. [file 12913_2024_10851_MOESM1_ESM.zip › Interview guide_patients.pdf]

Interview guide for individual interviews with patients within the trial “**Co**mmunication, **Co**ordination and **S**ecurity for Persons with **M**ultiple **S**clerosis” (COCOS-MS)

Introduction

As part of the COCOS-MS study, you were in regular contact with a care and case manager. Today I would like to talk to you about your previous personal experiences with the care and case manager and find out to what extent your care and everyday life have changed as a result. Although a trained scientific assistant also regularly conducted questionnaires with you, in today's interview you will have the opportunity to share your own personal experiences beyond that with us. This is very important for the comprehensive evaluation and further development of such a service.

Transition question (Directing to the topic) (5minutes)

Based on your own **personal experience** with the care and case manager, what would you say characterizes the work of a CCM to you?

Key questions (15minutes each)

**Opening question / narrative prompt**	**Memo for possible follow-up questions – only to be asked in case they are not addressed prior**	**Specific questions - ask in this wording**
**Block I: Functions and support of CCM**		

According to the definition, the work of a care and case manager can be divided into three key functions. On the one hand, this includes supporting people in safeguarding their own interests and managing their own affairs independently. On the other hand, mediating between the required service providers and the person, thus creating the appropriate access to care services. Based on this knowledge and your personal experience, what is your impression of the functions of a care and case manager? What tasks did the care and case manager take on for you (and your caregiver)?	- Identification and satisfaction of individual, illness-related needs<br>- Flexible response to changing needs<br>- Cross-sectoral approach to dealing with needs<br>- Unfulfilled wishes and how to deal with them<br>- Initiation or coordination of necessary services<br>- Person of trust / reliable contact person?<br>- Process control<br>- Relief?	- Can you describe to me if and how the care and case manager recognized your individual needs and advocated for your individual and changing concerns? Are there any examples you can give?<br>- At what point did the care and case manager not succeed or what would have to change in order to recognize or meet your needs even better? Please give examples.<br>- Please describe to me what influence, if any, the care and case manager had on establishing contact with professionals in the healthcare system, e.g. your doctors or therapists.<br>- You have just mentioned this, can you tell me more about this? To what extent and in what way were you able to experience relief from the care and case manager as a result of this or any other activities (if applicable, give examples such as arranging social law matters, finding suitable professional helpers, arranging appointments, general exchange of information)? What aspects remain open / unfulfilled to you?<br>- In what instances did you have the impression that you needed more support?
**Block II: Impact of the CCM intervention**<br><br>If you remember the time prior to taking part in the study, when the care and case manager did not yet visit or contact you regularly, what has changed for you in comparison, if anything?	- Benefit?<br>- Improvement?<br>- No change?<br>- Burden?<br>- Increase in quality of life?<br>- Strengthening of autonomy / co-determination?	- To what extent has your illness situation or your everyday life changed (positively or negatively) as a result of contact with the care and case manager?<br>- What effects (positive or negative) have you been able to perceive on yourself and your environment, e.g. on your relatives / friends / doctors / therapists, as a result of the care and case manager? Please describe them.

		If the patient cannot think of anything, ask specifically about the aspects in the memo
**Block III: Communication and evaluation**<br><br>If you now think of the first and last contact with the care and case manager, how did you feel about your first and last conversation with the care and case manager?	- Clarifying discussion in initial meeting<br>- Form of communication<br>- Opportunity to present own view of problems and solution strategies for current needs?<br>- Goals achieved?	- Can you describe the communication between you and the care and case manager? How did you feel about it?<br>- Which form of communication was more important to you, the weekly telephone calls or the monthly home visits?<br>- How or in what way were your expectations met?<br>- Are there any wishes or questions left unanswered?

**Closing Question** (10min)
What was most important to you about your contact with the care and case manager?<br>Is there anything else about care and case management that has not been mentioned but that you would still like to comment on?
